# Supplementary material for: Assessing Permutationally Invariant Polynomial and Symmetric Gradient Domain Machine Learning Potential Energy Surfaces for H3O2–
Source: J Phys Chem A. 2024 Apr 16;128(16):3212–9. doi: 10.1021/acs.jpca.4c01044 (PMC11056970; doi:10.1021/acs.jpca.4c01044)
Supplement: Supplementary file 1 — jp4c01044_si_001.pdf [file jp4c01044_si_001.pdf]

Supporting Information: Assessing PIP and sGDML Potential Energy Surfaces for $H_3O_2^-$

Priyanka Pandey,[†] Mrinal Arandhara,[‡] Paul L. Houston,[*,¶] Chen Qu,[§] Riccardo Conte,[∥] Joel M. Bowman,[*,†] and Sai G. Ramesh[*,‡]

†*Department of Chemistry and Cherry L. Emerson Center for Scientific Computation, Emory University, Atlanta, Georgia 30322, U.S.A.*

‡*Department of Inorganic and Physical Chemistry, Indian Institute of Science, Bangalore 560012, India*

¶*Department of Chemistry and Chemical Biology, Cornell University, Ithaca, New York 14853, U.S.A. and Department of Chemistry and Biochemistry, Georgia Institute of Technology, Atlanta, Georgia 30332, U.S.A*

§*Independent Researcher, Toronto, Toronto, Ontario M9B0E3, Canada*

∥*Dipartimento di Chimica, Università degli Studi di Milano, 20133 Milano, Italy*

E-mail: plh2@cornell.edu; jmbowma@emory.edu; sairamesh@iisc.ac.in

Table S1: Cartesian coordinates of the optimized global minimum, bifurcation TS, and H-transfer TS (in Angstrom), obtained from the PIP PES trained on all data.

Atom	Global Minimum			TS Bifurcation			TS H-transfer		
	x	y	z	x	y	z	x	y	z
O1	-1.2389	-0.0503	-0.0299	-1.3307	-0.0003	-0.0014	-1.2151	-0.0516	-0.0359
O2	1.2425	0.0454	-0.0389	1.2807	0.0018	-0.0352	1.2134	0.0501	-0.0385
H3	-0.1541	-0.0346	-0.0431	-0.6476	-0.6917	0.0243	-0.0008	-0.0007	-0.0424
H4	-1.4424	0.7066	0.5222	-0.6487	0.6920	0.0274	-1.4629	0.6590	0.5598
H5	1.5342	-0.6161	0.5944	1.9834	0.0009	0.6214	1.4626	-0.6613	0.5557

Table S2: Cartesian coordinates of the optimized *cis* and *trans* HO−OH torsion barriers (in Angstrom), obtained from the PIP PES trained on all data.

Atom	TS *cis*			TS *trans*		
	x	y	z	x	y	z
O1	-1.2471	-0.0515	-0.0011	-1.2435	-0.0641	0.0018
O2	1.2528	-0.0542	0.0017	1.2434	0.0618	0.0013
H3	-0.1722	-0.0427	0.0001	-0.1637	-0.0456	0.0016
H4	-1.4673	0.8813	-0.0014	-1.4689	0.8676	0.0009
H5	1.5582	0.8574	0.0019	1.6008	-0.8299	0.0022
